# PSMA-targeting agents for radio- and fluorescence-guided prostate cancer surgery

**DOI:** 10.7150/thno.36739

**Published:** 2019-09-20

**Authors:** Yvonne H.W. Derks, Dennis W. P. M. Löwik, J. P. Michiel Sedelaar, Martin Gotthardt, Otto C. Boerman, Mark Rijpkema, Susanne Lütje, Sandra Heskamp

**Affiliations:** 1 Department of Radiology and Nuclear Medicine, Radboud University Medical Center, Nijmegen, The Netherlands; 2 Radboud University Nijmegen, Institute for Molecules and Materials, Systems Chemistry, Nijmegen, The Netherlands; 3 Department of Urology, Radboud University Medical Center, Nijmegen, The Netherlands; 4 Department of Nuclear Medicine, University Hospital Bonn, Bonn, Germany

**Keywords:** PSMA, image-guided surgery, radionuclide, fluorescence, multimodal imaging.

## Abstract

Despite recent improvements in imaging and therapy, prostate cancer (PCa) still causes substantial morbidity and mortality. In surgical treatment, incomplete resection of PCa and understaging of possible undetected metastases may lead to disease recurrence and consequently poor patient outcome. To increase the chance of accurate staging and subsequently complete removal of all cancerous tissue, prostate specific membrane antigen (PSMA) targeting agents may provide the surgeon an aid for the intraoperative detection and resection of PCa lesions. Two modalities suitable for this purpose are radionuclide detection, which allows sensitive intraoperative localization of tumor lesions with a gamma probe, and fluorescence imaging, allowing tumor visualization and delineation. Next to fluorescence, use of photosensitizers may enable intraoperative targeted photodynamic therapy to eradicate remaining tumor lesions. Since radiodetection and optical imaging techniques each have their own strengths and weaknesses, a combination of both modalities could be of additional value. Here, we provide an overview of recent preclinical and clinical advances in PSMA-targeted radio- and fluorescence-guided surgery of PCa.

## Introduction

Despite recent improvements in imaging and therapy, PCa remains the most frequently diagnosed cancer type in men and is estimated to be the second leading cause of cancer related deaths 1, 2. Currently, PCa treatment is dependent on the stage of the disease at initial diagnosis. Possible early stage management consists of active surveillance or watchful waiting. However, if treatment is deemed necessary, due to progression of the disease under active surveillance, or if the initial stage of the disease mandates immediate treatment, curative options are indicated. One of these curative options is surgical removal of all cancerous tissue: radical prostatectomy with or without pelvic lymph node dissection 3. Depending on the extent of the disease, surgery may be combined with adjuvant radiotherapy, hormone therapy and chemotherapy, especially in patients with high risk PCa, lymph node positive disease or positive surgical margins after resection 3.

Surgical treatment of PCa faces two main challenges. First, complete resection of the prostate tumor, and thus managing negative surgical margins, remains difficult 4. While removal of the entire prostate gland makes complete resection of the primary tumor feasible, it often leads to nerve damage that may cause debilitating functional side effects such as urinary incontinence and erectile dysfunction. Therefore, surgeons are often on the proverbial knife edge between complete oncological resection and nerve-saving operations with a maximum chance of good functional outcome, but a higher chance of positive resection margins as a consequence 4. The average rate of positive surgical margins after radical prostatectomy is 15% and can increase up to 50% in men with more locally advanced disease 4, 5. Hence, margin detection is of utmost importance for the surgical management and clinical outcomes of PCa patients. The second main challenge is the detection of tumor positive lymph nodes visualized in preoperative ^68^Ga/^18^F-PSMA PET or nano-MRI scans 4, 6, 7. The ability to find and resect metastatic lymph node tissue is currently based on anatomical landing sites like the obturator region together with the external and internal ileac artery region. Most small nodes are invisible for the surgeons eye, and are not palpable during surgery 7. Intraoperative tissue shifts, atypical locations, small size and inconspicuous morphology hamper the detection of tumor positive lymph nodes during surgery and thus their resection 6, 7. In addition, metastatic tumor lesions can be difficult to resect due to proximity to other tissue (nerves, blood vessels, bladder and rectum) 4. The effect of incomplete resection can be profound for the individual patient as it may lead to early disease recurrence and poor patient outcome 8, 9. To increase the chance of complete resection of all tumor tissue, more sensitive and specific techniques are needed that allow for intraoperative real time detection of all cancer tissue including the tiniest lesions.

In order to detect PCa, prostate-specific membrane antigen (PSMA), a type II transmembrane glycoprotein, represents an excellent target for several reasons. Firstly, PSMA is over-expressed on >90% of all primary PCa lesions, as well as tumor positive lymph nodes and distant metastasis. In contrast, PSMA expression on healthy tissues is minimal 10-12. Secondly, PSMA expression in PCa correlates with the Gleason grading of the prostate lesions, with high expressions in high Gleason scores 13. Finally, binding of a targeting agent to the active center of the extracellular domain of PSMA generally leads to internalization of the agent, resulting in enhanced retention of the label in the lesion 6. Due to these properties, PSMA constitutes a reliable molecular marker for PCa and is an ideal target for imaging and therapy of PCa. So far, a wide variety of PSMA-targeting agents have been described, varying from intact antibodies to low-molecular-weight compounds 14. Small molecules exploit the binding of substrates, including N-acetyl-L-aspartyl-L-glutamate (NAAG) and folate (poly)gamma glutamate substrates, to the active site of PSMA 15, 16. First, glutamate containing phosphoramidates or 2-(phosphinylmethyl) pentanedioic acid based PSMA-binding motifs were synthesized 17, 18. Later, glutamate-urea-lysine based PSMA-binding motifs were developed and are now most frequently used 19. The latter compounds are structurally more similar to NAAG and therefore exhibit the best PSMA-binding properties 20.

Pre- and intraoperative identification of malignant tissue could be improved using multiple techniques including radioguidance, fluorescence imaging, ultrasound- and/or MRI-guidance. In this review we will focus on and discuss the potential of the currently available PSMA-targeting agents for radio-, fluorescence- and multimodal-guided intraoperative detection of PCa lesions. In addition, the feasibility of near infrared (NIR) photosensitizer-conjugated PSMA-tracers for intraoperative targeted photodynamic therapy (tPDT) is discussed.

## PSMA-radioguided surgery in PCa

Over the past five years, the intraoperative PSMA-radioguided surgery (RGS) approach entered the operating theater for PCa patients undergoing prostatectomy with or without extended pelvic lymphadenectomy. Radioguided lymph node dissections were applied in an experimental setting in patients both within the typical field of an extended lymph node template (iliaca externa, interna, communis, obturator fossa) and patients with atypically localized lesions (retrovesical/seminal, presacral/pararectal, retroperitoneal) 21-23. RGS is based on the detection of γ-photons with handheld probes. These γ-photons have virtually infinite penetration through human tissue. Consequently, intraoperative detection of radionuclides is generally not hindered by penetration depth and is considered highly sensitive 24. The above mentioned properties of RGS make this technique highly suitable to tackle the second challenge in PCa surgery; incomplete detection of tumor positive lymph nodes. Despite the high sensitivity, the intraoperative use of radionuclides remains limited as surgeons need to rely on an acoustic signal and exact visualization of the tumor tissue is lacking, which may cause inaccurate tumor delineation 24. Moreover, due to the high penetration depth of background signals from tracers accumulating in other organs accurate detection can be hampered. For instance, detection in the vicinity of the kidneys is impossible for tracers with high kidney retention, the same goes for the bladder 6. Intraoperative radionuclide imaging using a handheld SPECT camera is currently not commonly used as an imaging method, as its value is limited by a low spatial resolution, lack of anatomical reference and relatively high radiation exposure 14.

PSMA-based SPECT/CT and PET/CT can accurately detect primary PCa and small lymph node metastasis up to a size of 0.5-1 cm^3^ preoperatively 6. However, during surgery, reliable identification of small and/or atypically localized lesions can be challenging which can lead to incomplete resection of lymph node metastasis or extensive removal of non-affected lymph nodes. Recent studies investigated the precision of salvage lymph node dissection (sLND), a still controversial surgical approach for PCa patients 25-27. In this sLND approach the dissection field and removal of lymph nodes is based on well-defined surgical regions in combination with advanced imaging such as preoperative ^68^Ga/^18^F-PSMA-PET imaging. Among patients that undergo sLND a biochemical response is reported in 40-60% of the patients and a complete biochemical response is only observed in 20-30% of the patients. One of the reasons for these disappointing results could be the incomplete surgical resections of all involved tumor positive lymph nodes 25, 27. In another study, final pathology revealed no metastases in lesions removed based on preoperative PET/CT imaging in 31% of the patients with conventional surgery, despite the removal of on average 13.6 (range: 5-27) lymph nodes per patient 26. Hence, the surgical treatment for patients with PCa needs improvement. This might be achieved by combining preoperative radionuclide imaging with intraoperative radionuclide detection. For example, many resections involve significant tissue movements and deformation, making preoperative scan-based navigation imprecise 6. For the surgeon, the combined knowledge from the preoperative scans and real-time feedback during RGS might therefore provide vital information on the location, the extend of the disease and positive surgical margins. Moreover, it may aid the surgeon in minimizing the invasiveness of the surgical procedure 28.

In PCa surgery, radioguided resection using ^99m^Tc-nanocolloid was first introduced for sentinel lymph node dissections. After injection in the prostate, the ^99m^Tc-nanocolloid distributed to lymph nodes along the internal and external iliac vessels and the obturator fossa 29. Even though this technique does not use a PSMA-targeted tracer, it shows the potential of radionuclide-guided resection 30, 31. The effectiveness of radioguided sentinel lymph node dissections was tested in a large study in 2,000 patients by Holl *et al*. 29. They reported a detection rate of 98%. Nonetheless, in 24% of the cases, not all preoperatively visualized nodes could be removed during surgery, presumably due to nodal uptake as low as 0.07% injected dose, leading to a count rate too low for intraoperative detection of the node 32, 33. Of the entire number of SLN (n=12141, in 2020 patients) resected only 600 (4.9%) were positive for metastases, indicating the need for more specific cancer detection strategies in radioguided resection of PCa and its metastasis 29. Nonetheless, accuracy of sentinel lymph node procedures in PCa could be improved, for instance with the use of a hybrid indocyanine green-^99m^Tc-nanocolloid (radioactive and fluorescent) 31.

**Figure 1:**
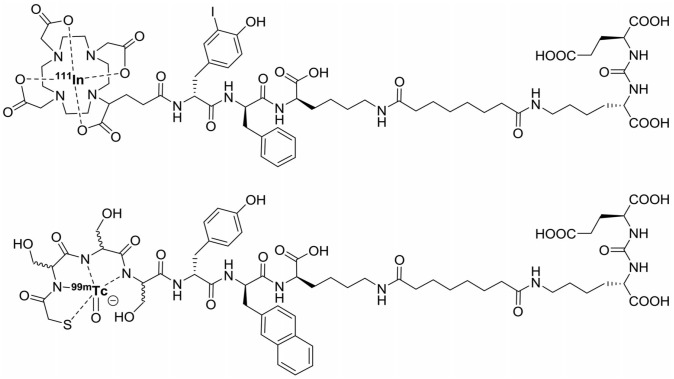
Chemical structures of [^111^In]In-DOTAGA-(3-iodo-y)-f-k-Sub(KuE) (PSMA-I&T) and [^99m^Tc]Tc-mas3-y-nal-k(Sub-KuE) (PSMA-I&S).

With the introduction of PSMA-targeted RGS (PSMA-RGS) detection of both primary and distant PSMA-positive tumors was made possible 6. PSMA-RGS makes use of radiolabeled PSMA-targeting agents such as antibodies, peptides, and small molecules 21, 23, 34-36. Use of PSMA-targeting tracers for RGS potentially allows the detection of tiny and atypically located lesions 6. In a PSMA-RGS feasibility study, Maurer *et al.* used the [^111^In]In-DOTAGA-(3-iodo-y)-f-k-Sub(KuE) (^111^In-PSMA-I&T, Figure 1) small molecule ligand to evaluate PSMA-RGS for the detection of metastatic PCa lesions 35. Five patients received an intravenous injection of 124 MBq ^111^In-PSMA-I&T, 24 hours before surgery. Metastatic tissue was tracked intraoperatively using a gamma probe and all tissues that showed radiosignal were removed. The presence of tumor tissue was confirmed by *ex vivo* histopathology.

**Figure 2:**
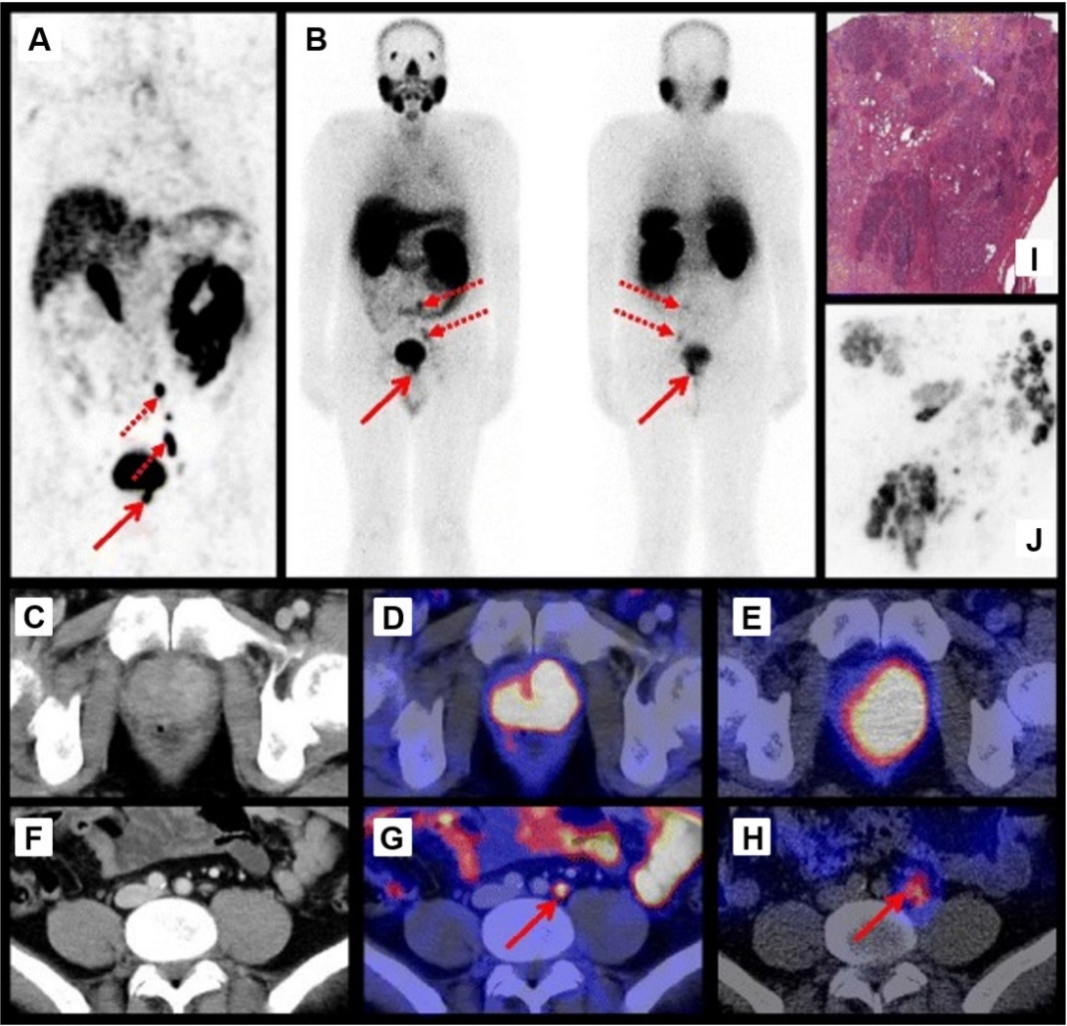
Preoperative imaging using ^68^Ga-PSMA-11 PET/CT 1 h p.i. **(A)** and ^111^In-PSMA-I&T SPECT/CT and planar scintigraphy (4 h p.i., 155 MBq) **(B)**. Axial ^68^Ga-PSMA-11 PET/CT images of the primary tumor in the prostate **(D)** and a representative lymph node **(G)**. Corresponding CT images **(C, F)** and axial ^111^In-PSMA-I&T SPECT/CT images **(E, H)**. H&E staining **(I)** and ^111^In-autoradiography **(J)** of cryosections from resected prostate tissue. The human study was approved by the institutional review boards of the participating medical institutions, and the patient provided signed informed consent. Reprinted with permission from Schottelius et al., ^111^In-PSMA-I&T: expanding the spectrum of PSMA-I&T applications towards SPECT and radioguided surgery, Copyright 2015, Springer 22.

Maurer *et al.* demonstrated that ^111^In-PSMA-I&T based RGS allowed the resection of subcentimeter lesions and led to the removal of additional PCa lesions. In 2 patients positive lesions, as confirmed by histopathological analysis, were found that did not show up during preoperative [^68^Ga]Ga-HBED-CC-PSMA (^68^Ga-PSMA-11) PET/CT imaging 35. Schottelius *et al*. also successfully applied the ^111^In-PSMA-I&T ligand for radioguided resection of PSMA-positive lesions in an exemplary PCa patient (Figure 2A-J) 22. Both studies demonstrate the potential of ^111^In-PSMA-I&T ligand for intraoperative detection of tumor positive lymph nodes in PCa. More recently, Robu *et al.* explored the potential of [^99m^Tc]Tc-mas3-y-nal-k(Sub-KuE) (^99m^Tc-PSMA-I&S, Figure 1) mediated PSMA-RGS in two exemplary PCa patients 21. As the half-life of ^99m^Tc is much shorter than that of ^111^In (6 hours *vs*. 2.8 days), timing of the surgery is crucial. However, the low energy gamma rays produced by ^99m^Tc could be advantageous for RGS, as gamma probes have the highest sensitivity for predominantly lower energy gamma photon emitting radionuclides 28. In all suspected lesions previously identified by ^68^Ga-PSMA-11 PET/CT imaging, a high uptake of ^99m^Tc-PSMA-I&S was observed 12 hours after injection, as measured by preoperative SPECT/CT imaging. This high uptake resulted in successful intraoperative detection and resection of radiosignal-positive lesions 21. In a retrospective analysis Maurer *et al.* compared the radioactive rating (positive *vs*. negative) of tissues resected during ^99m^Tc-PSMA-I&S-RGS to their postoperative histopathological analysis 23. Tissue specimens were considered positive if a count rate of at least twice the background was measured. The radioactive rating yielded an accuracy of 93% (confidence interval [CI]: 85.5-96.7%), a sensitivity of 83.6% (CI: 70.9-91.5%), and a specificity of 100%. During the ^99m^Tc-PSMA-I&S-RGS all tumor lesions visualized on ^68^Ga-PSMA-11 PET could be resected and additional small metastasis, down to 3 mm in size, could be removed 23. In all patients a urinary catheter was inserted that allowed for removal of radioactive urine from the bladder, which otherwise could impair gamma probe measurements. In approximately one third of the patients treated with salvage PSMA-RGS, surgery related complications were noted, mostly consisting of a (temporary) worsening of incontinence, bladder leakage or lymphedema, which also occur during conventional surgery 23, 24, 36.

Subsequent to the above mentioned studies multiple clinical follow up studies were performed using either ^111^In-PSMA-I&T or ^99m^Tc-PSMA-I&S mediated PSMA-RGS. In these studies PSA levels and time to biochemical recurrence were monitored 23, 24, 36. However, no comparison was made to the conventional surgical treatment of PCa. So far, no randomized trials have been performed that compare PSMA-RGS with a conventional surgical approach, in which the dissection field is based solely on preoperative ^68^Ga/^18^F-PSMA-PET imaging. However, designing these studies is challenging as surgical fields are difficult to define, outcomes of surgeries are hard to compare due to inter-surgeon quality differences, and the long survival of the patients hampers fast oncological outcome measurements. Nonetheless, larger patient numbers and long-term follow-up studies are needed to determine the clinical benefit of PSMA-RGS. These studies should include comparison of treatment-related complications, progression-free survival, overall survival and quality of life of PCa patients.

Taken together, PSMA-RGS still represents an individual and not guideline-conform treatment concept without proven benefit on quality of life or cancer-specific survival, despite the large quantities of proof of principle data 37. Nevertheless, PSMA-RGS is feasible and is an aid for the surgeon to achieve complete resection of all PCa lesions using audio-based intraoperative and *ex vivo* gamma-probe measurements. Ultimately, this might lead to improved oncological outcomes. Still, use of radioguidance could only solve one of the two major challenges in PCa surgery. Even though it might improve detection and removal of tumor positive lymph nodes, it is not an optimal aid for the surgeon in visualization and precise delineation of the primary tumor and hard-to-resect metastasis.

## PSMA-targeted intraoperative NIRF imaging

In recent years, an alternative intraoperative imaging method, near infrared fluorescence (NIRF) imaging, has entered the surgical theatre. Fluorescence imaging might be used to tackle the first challenge in the surgical treatment of PCa: achieving negative surgical resection margins 38. It relies on fluorescent markers to accumulate in malignant tissue and produce optical photons upon absorption of light of a specific wavelength, which can in turn be detected by a fluorescence camera system 39. Normal tissue exhibits very low auto-fluorescence in the NIR spectrum, resulting in high signal to background ratios. With NIR imaging techniques, tumor lesions can be directly visualized and clearly distinguished from healthy tissue 39, 40. Other advantages of fluorophores are that they can be excited repeatedly, have extended half-lives and have a high spatial and temporal resolution 38. However, the vast majority of the photons produced by NIR fluorophores will be scattered or absorbed within a few cell layers of tissue 40. This means that fluorescence imaging is limited by low tissue penetration depth 41. Another essential challenge is the lipophilicity of many NIR fluorophores, which may significantly change the pharmacokinetics and tumor targeting properties of the targeting ligands 42.

**Figure 3:**
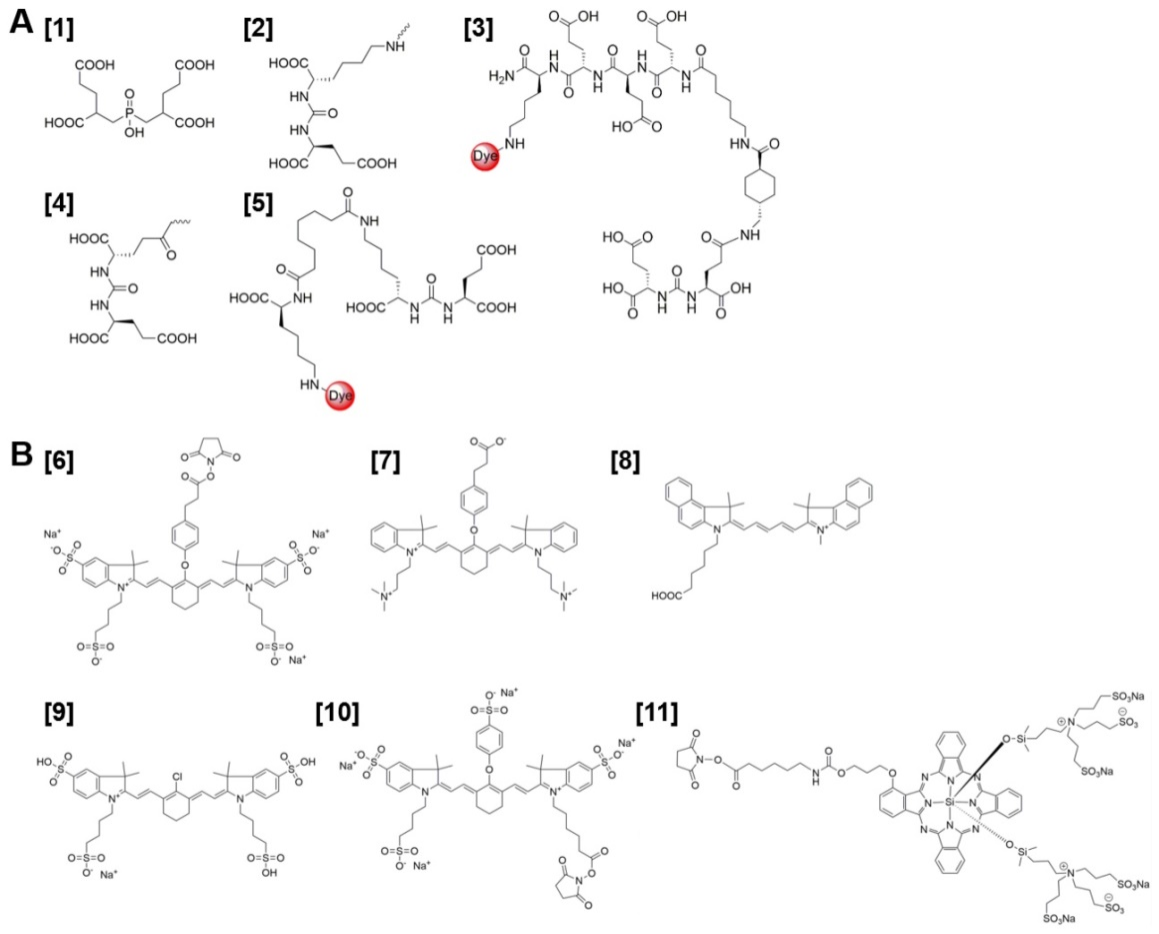
** (A)** Chemical structure of PSMA-binding motifs GPI **[1]**, glutamate-urea-lysine (KuE) **[2]**, PSMA-1 **[3]**, DUPA **[4]**, and YC27 **[5]**. **(B)** Chemical structures of fluorescent dyes IRDye78 **[6],** ZW800+3C **[7]**, Cy5.5 **[8]**, SO456 **[9]**, IRDye800CW **[10]**, IRDye700DX **[11]**.

PSMA-targeted NIRF imaging is emerging as an attractive strategy for visual guidance during PCa surgery. The success of this strategy largely depends on the production of PSMA-targeted NIRF probes with optimal *in vivo* targeting characteristics. A series of PSMA-targeted contrast agents have been developed by conjugating different NIR fluorophores to PSMA-targeting whole antibodies, antibody fragments and small molecules 34, 43, 44. The first small molecule PSMA-NIR fluorophore conjugates were produced by Humblet *et al*. who conjugated the highly potent PSMA inhibitor GPI **[1]** to IRDye78 **[6]** to create the molecule GPI-78 (Figure 3A-B). Interestingly, the NIR fluorophore conjugation enhanced PSMA binding affinity over 20-fold. According to the authors this was due to the four sulfonic acid groups present in the IRDye78 that also show PSMA binding affinity 43. This study emphasizes a main challenges of using small PSMA binding motifs for NIR-dye conjugation; varieties in imaging moieties and their linkers can lead to major differences in affinity, pharmacokinetics and tumor uptake of the tracer. This does not necessarily have to be a disadvantage. For example, an increase in the length of the linker was described to increase affinity for PSMA and the addition of multiple negative charges showed improved tumor-to-background ratios 45-48. Bao *et al*. systematically conjugated different NIR fluorophores to the glutamate-urea-lysine PSMA binding motif **[2]** 34. The PSMA binding motif coupled to the ZW800+3C fluorophore **[7]**, with a linker length of 18 Å and a net charge of minus 6, showed optimal *in vitro* and *in vivo* PSMA binding. In addition to the effects of the linker, the physicochemical properties of conjugated fluorophores can also drastically alter characteristics of the small PSMA-targeting molecules. Changes were observed not only in hydrophobicity and polarity but also in binding affinity 34, 43, 44. Wang *et al*. synthesized a high-affinity PSMA ligand called PSMA-1 **[3]** and labeled it with NIR dyes IRDye800 and Cy5.5 **[8]**. These PSMA-1-NIR probes were shown to selectively target orthotopic PSMA-positive PC3-PIP tumors and allowed intraoperative tumor visualization using NIRF-imaging. Their results indicated that the pharmacokinetics of the probes were highly dependent on the type of fluorophore conjugated to the PSMA-1 ligand. These differences in pharmacokinetics were observed in blood clearance, tumor retention, liver and kidney uptake. Overall, the most hydrophilic and negatively charged dyes cleared fastest 44. Another property that should be taken into account is the lipophilicity of a fluorophore, which affects binding properties and internalization of the tracer and thereby its retention in the tumor 49.

**Figure 4:**
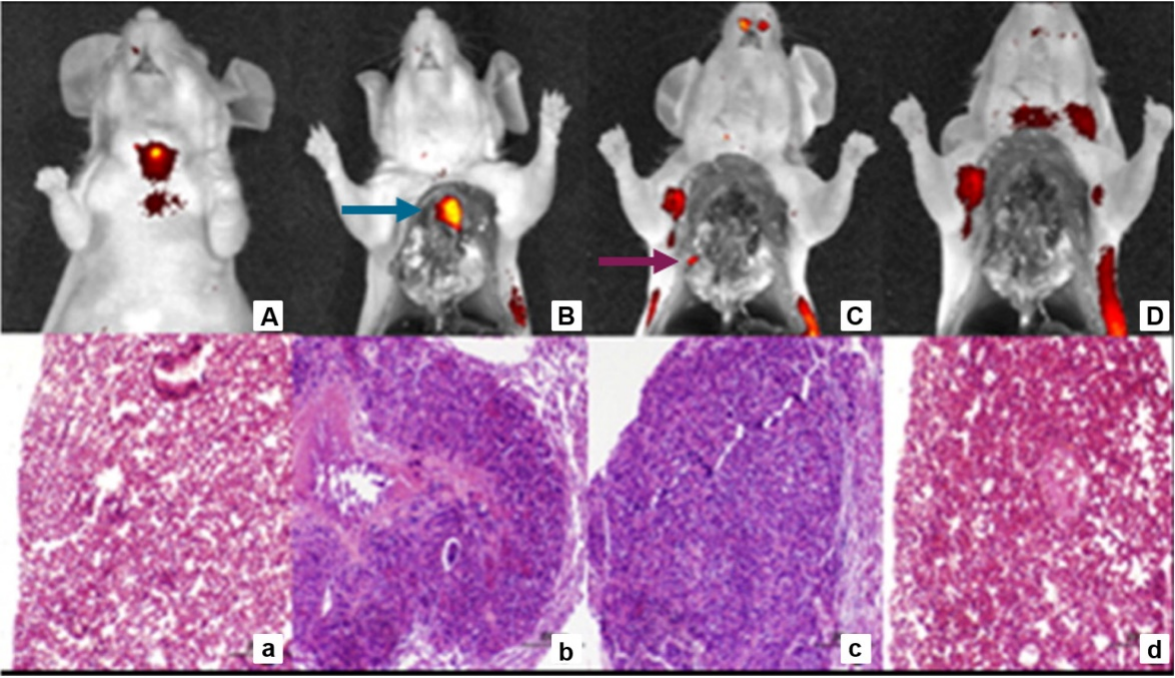
Sequential tumor debulking surgery and H&E analysis of 22RV1 tumor metastases 4 h p.i. with DUPA-IRDye800CW (10 nmol). Fluorescent and white light image overlays of whole body image **(A)**, opened chest cavity **(B)**, after the removal of the primary tumor (blue arrow) **(C)** and after the removal of all secondary nodules (purple arrow) **(D)**. H&E staining healthy control lung **(a)**, primary tumor **(b)**, secondary tumor nodule **(c)** and residual tissue **(d)**. Reprinted with permission from Kelderhouse et al., Development of tumor-targeted near infrared probes for fluorescence guided surgery, Copyright 2013, American Chemical Society 52.

Despite the fact that attachment of NIR dyes could alter small molecule PSMA-binding properties, NIR-conjugated ligands already showed high potential in multiple preclinical studies. However, it should be noted that the *in vivo* tumor targeting properties of the different NIR-conjugated ligands cannot be directly compared, as PSMA expression levels differ among the various tumor models used. For example, uptake of [^177^Lu]Lu-DOTA-PSMA-617 tracer varied between 8 %ID/g and 44 % ID/g for LNCaP and PC3-PIP xenografts respectively 48, 50. Kularatne *et al*. developed a NIR tracer using a small molecule PSMA inhibitor called DUPA **[4]** and the S0456 NIR dye **[9]** 51. This tracer was shown to avidly bind PSMA positive tumors with high specificity in mice. The fluorescent signal in PSMA-positive tumors lasted for more than 48 hours, allowing visualization throughout fluorescence-guided surgery 51. Moreover, Kelderhouse *et al*. developed PSMA-specific probes by combining fluorophores like Alexafluor 647, Dylight680 and IRDye800CW **[10]** with the PSMA inhibitor DUPA **[4]** 52. Tracers were tested in a metastatic mouse model. Intravenous injection of these PSMA-ligand NIR dye tracers enabled fluorescence imaging of the PSMA-expressing metastases, allowing their complete resection with minimal contamination of surrounding healthy tissues. The intraoperative resection of these tumor lesions was performed in stages, meaning that larger lesions were resected first. Importantly, excision of these more prominent fluorescent masses often revealed smaller lesions that could have been missed without the aid of PSMA-specific fluorescence (Figure 4A-D) 52. Another low-molecular weight urea-based fluorescent PSMA binding agent called YC-27 **[5]** was conjugated to the NIR dye IRDye800CW **[10]**.

Kovar *et al*. described that YC-27-800CW showed good tissue contrast and tumor delineation at doses as low as 0.25 nmol 1. Subsequently, Neuman *et al.* used a PCa murine model with subcutaneous PC3-PIP PSMA-expressing tumors to perform image-guided resection of tumor lesions 53. In 8 mice tumors were resected with the guidance of a NIRF imaging system, while the remaining animals (n = 10) were resected under normal white light. None of the animals resected with YC-27 fluorescence-guidance developed a recurrence, whereas 40% of the mice with conventional white light resection developed recurrence within 30 days after resection 53. Together, these studies suggest that PSMA-binding small molecules or antibody fragments coupled to NIR dyes have the potential to be further developed for clinical use as contrast agents in fluorescence-guided surgery. However, the fact that coupling of the NIR dye can drastically alter the properties of the contrast agent should be taken into account during development of such a tracer. Nevertheless, it is difficult to predict the *in vivo* properties of these ligands consisting of multiple structural parts that often differ between tracers.

## Best of both worlds: PSMA-targeted multimodal image-guided surgery

**Figure 5:**
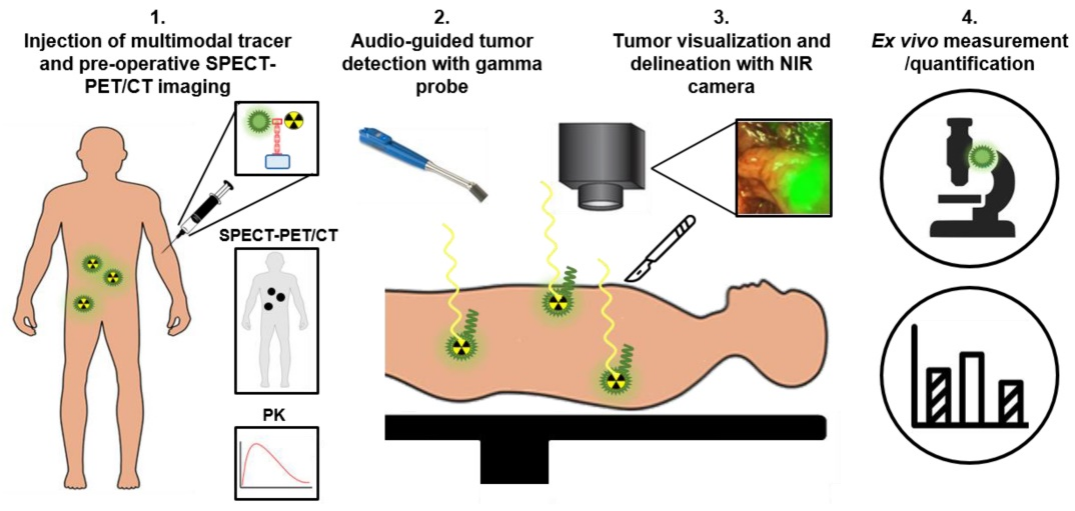
Schematic representation of multimodal radio- and fluorescence-guided surgery. PK: pharmacokinetics

To overcome the specific drawbacks of the radionuclide and fluorescence-imaging modalities, the focus of intraoperative PSMA-targeted tracer research has shifted towards combining both fluorescence- and radionuclide detection techniques for PCa surgery 5, 54-56. In case of PCa, multimodal PSMA-targeting agents potentially can be used for four applications (Figure 5). First, the radionuclide allows preoperative PET/CT or SPECT/CT imaging to localize all tumor tissue. Second, a handheld gamma probe can be used to localize both deep-seated and superficial metastatic tumor lesions intraoperatively based on the radiosignal, guiding the surgeon until the fluorescent signal becomes visible. Due to its high penetration depth, radioguidance is especially suitable for the detection and guidance towards metastatic lesions, nonetheless no accurate real time visualization can be made. Therefore, third, the fluorescent label can be used to directly visualize the tumors during surgery, opening up the opportunity for resections without positive surgical margins. Fourth and final, an accurate quantitative assessment can be made to determine the pharmacokinetics of the tracer, to facilitate dose finding studies, and to perform *ex vivo* examination of resected tissue 14. By placing both radionuclide and fluorescent tags on the same tracer, potential complications associated with co-injected mixtures can be avoided, including receptor saturation due to limited receptor expression and differences in pharmacokinetics, biodistribution, or PSMA binding affinity 56.

Encouraging results were reported in several preclinical studies that have focused on the development of dual-labeled radionuclide and fluorescent PSMA-targeting agents. Lütje *et al*. evaluated the potential of multimodal image-guided surgery of PCa with the anti-PSMA monoclonal antibody D2B, labeled with both ^111^In and the NIR dye IRDye800CW (Figure 3B, **[10]**) 5. Two days after injection of the [^111^In]In-DTPA-D2B-IRDye800CW conjugate, intraperitoneal LS174T PSMA-expressing tumors could be visualized specifically with both imaging modalities. Subsequent image-guided resection showed the feasibility of complete multimodal-guided resection of all intraperitoneal tumor lesions *in vivo*. Interestingly, in a mouse with several LS174T-PSMA tumors located at different depths in the peritoneal cavity, the limited penetration depth of NIRF imaging was demonstrated. Only the two most superficial tumors could be visualized with NIRF imaging, whereas microSPECT/CT imaging showed several additional tumor lesions 5. However, the mAb D2B is a murine IgG and should at least be humanized before clinical translation is feasible, which is not the case for dual-labeled small molecule PSMA-ligands. Other advantages of using small-molecule multimodal tracers are fast tumor targeting and rapid blood clearance 11, 57.

**Figure 6:**
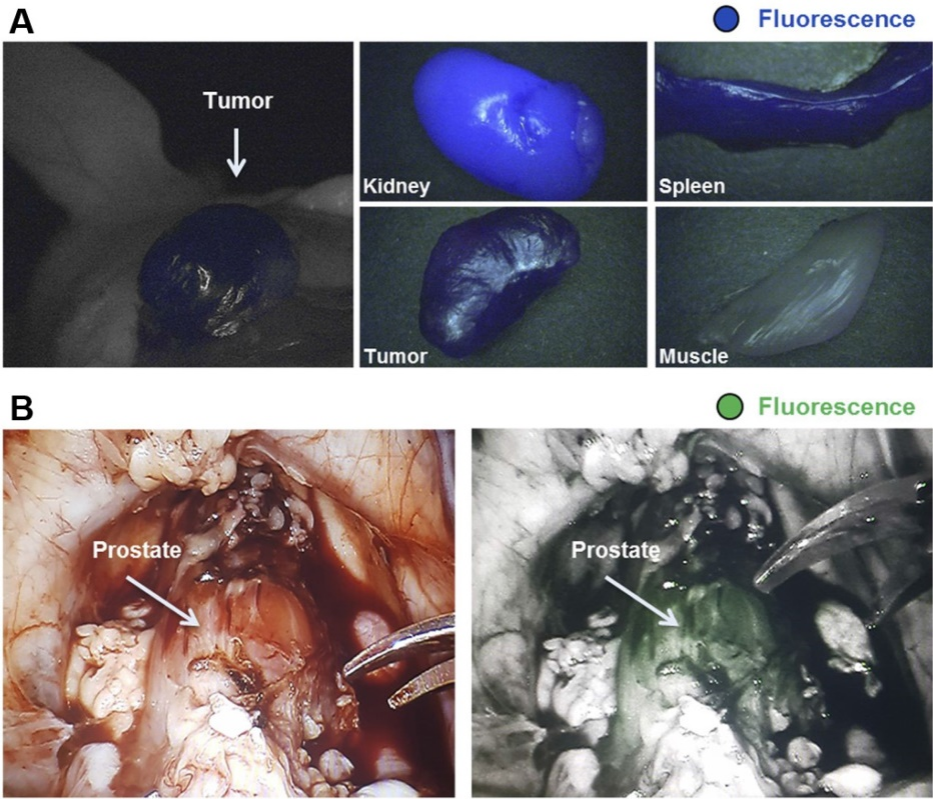
Proof-of-principle fluorescence-guided surgery studies with multimodal ^68^Ga-IRDye800CW-PSMA-11 in tumor-bearing mice and healthy pigs.** (A)** ^68^Ga-IRDye800CW-PSMA-11 (0.5 nmol) was injected in s.c. LNCaP tumor-bearing mice for small-animal PET imaging, followed by ex vivo fluorescence detection 2 h p.i. (IMAGE1 S system). **(B)** After preimaging acquisition of background fluorescence (da Vinci FireFly system), IRDye800CW-PSMA-11 (30 μg/kg) was injected i.v. in healthy pigs. 1 h p.i. fluorescence-guided prostatectomy using *in vivo* and *ex vivo* fluorescence detection was performed. This research was originally published in *JNM*, Baranski et al., PSMA-11-Derived Dual-Labeled PSMA Inhibitors for Preoperative PET Imaging and Precise Fluorescence-Guided Surgery of Prostate Cancer, J Nucl Med, 2017, © SNMMI 54.

In 2011, Banerjee *et al*. developed a dual-labeled, glutamate-urea based, PSMA-targeting SPECT/NIRF imaging small molecule tracer conjugated with IRDye800CW and a DOTA chelator, labeled with^ 111^In 58. They showed clear multimodal tumor visualization and high tracer uptake in subcutaneous PSMA-expressing PC3-PIP tumors (16.4±3.7 % ID/g), compared to the PSMA-negative PC3-flu control tumors (1.9± 0.2 % ID/g, 5 hours p.i.). Subcutaneous xenografts were delineated specifically, both with SPECT and NIRF imaging. However, intense tracer uptake was observed in the kidneys, which can be explained by the route of excretion of both tracers 58. Baranski *et al*. developed a series of PSMA-11 targeting ligands conjugated with different fluorophores, among which the NIR IRDye800CW and Dylight800 54. PSMA-specific uptake of the IRDye800CW (13.6 ± 3.7 %ID/g) and DyLight800 (15.6 ± 5.5 %ID/g) conjugated ligands in the tumor was significantly higher than that of the unconjugated ^68^Ga-PSMA-11 agent (4.8 ± 1.3 %ID/g). Furthermore, proof of concept fluorescence-guided surgery studies with ^68^Ga-IRDye800CW-PSMA-11were performed in healthy pigs using a DaVinci robotic surgery system. A PSMA-specific fluorescent signal (1 hour p.i. of the tracer) was observed in the prostate, which expresses PSMA on a physiological level. This demonstrated the potential of the ^68^Ga-IRDye800CW-PSMA-11 ligands for fluorescence-guided radical prostatectomy (Figure 6A-B) 54.

Very recently, Schottelius *et al*. developed a tracer named PSMA Imaging and Fluorescence (PSMA-I&F). Based on the previous PSMA-I&T, with addition of the fluorophore Sulfo‐Cy5. PSMA-specific tracer uptake into LNCaP xenografts (4.5 ± 1.8 %ID/g) led to sufficient imaging contrast in ^68^Ga-PSMA-I&F PET and in intraoperative fluorescence imaging 59. However, in both studies mentioned above, only the fluorescent signal was used for guidance during resection, providing the first proof of concept for multimodal image-guided resection. For future work, it would be interesting to use both detection modalities during surgery, as for instance has been done in renal cell carcinoma 60, to gain insight into the added value of combining both imaging modalities.

So far, these preclinical studies demonstrate the potential value of multimodal strategies for image guidance during surgery. However, translation of the PSMA-targeting multimodal preclinical tracers to the clinical setting still has to be made. For other malignancies, multimodal tracers were translated to the clinic, indicating the potential of multimodal treatments in patients. For example, Hekman *et al*. showed that tumor targeted multimodal imaging using [^111^In]In-DOTA-girentuximab-IRDye800CW is safe and can be used for intraoperative guidance in patients with clear cell renal cell carcinoma 60. Nonetheless, additional studies are needed to evaluate the additional value of multimodal image-guided surgery compared to conventional mono-modal surgery techniques for complications, quality of life and overall survival and patient outcome.

## Theranostics: Photodynamic therapy using photosensitizer-conjugated PSMA tracers

**Figure 7:**
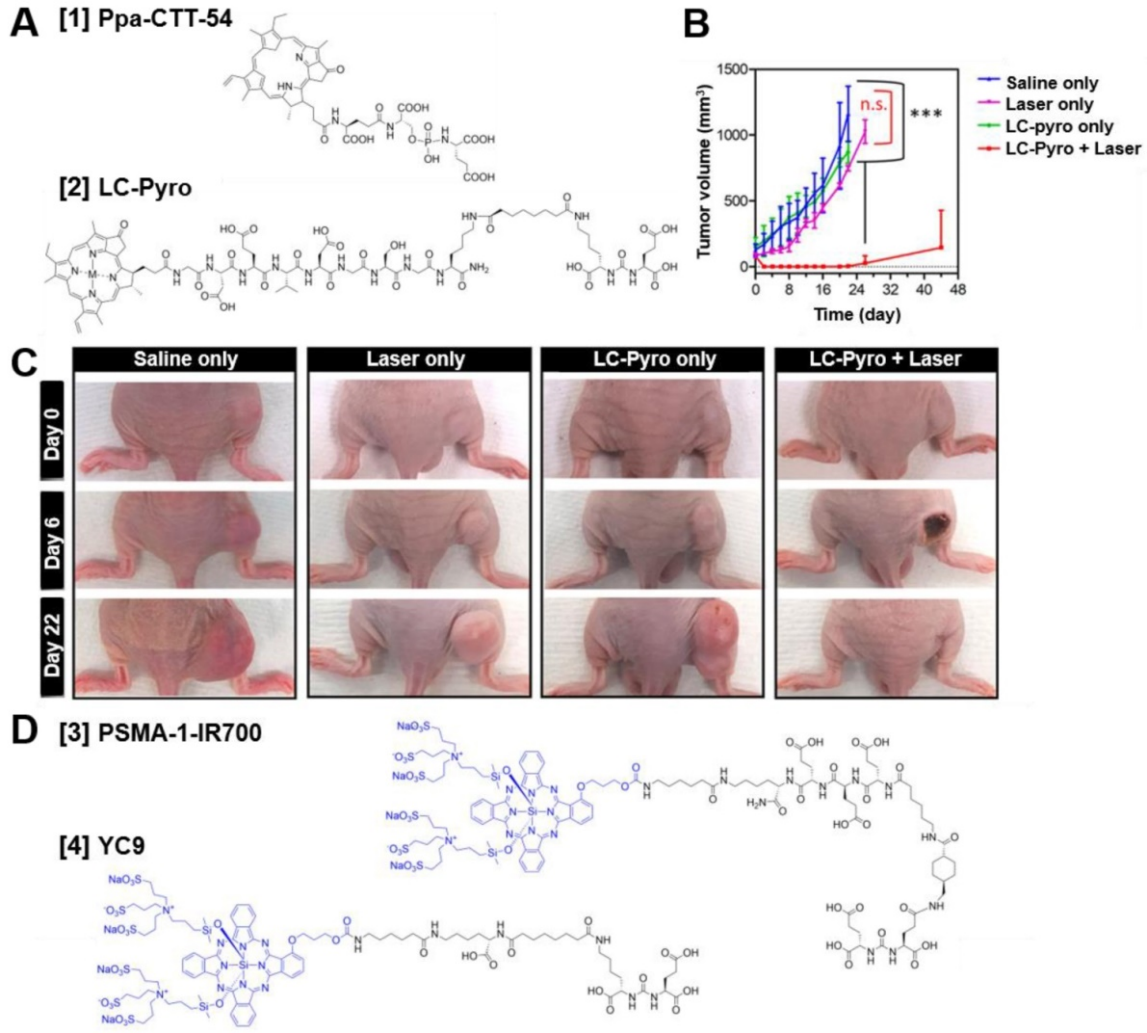
Chemical structures of different PSMA-targeted photosensitizer conjugates and example photodynamic therapy (PDT) efficacy of LC-Pyro *in vivo*.** (A)** Chemical structures of Ppa-CTT-54 **[1]** and LC-Pyro **[2]**. **(B)** PDT efficacy of LC-Pyro in PSMA^+^ PC3-PIP s.c. tumor-bearing mice. Tumor growth curves (mean ±SD, n = 4 for each group, ***P ≤ 0.001, n.s. = not significant). **(C)** Representative images of tumor-burdened mice in saline only, laser only, LC-Pyro only, and LC-Pyro + Laser groups at 0, 6, and 22 days post-PDT treatment. **(D)** Chemical structures of PSMA-1-IR700 **[3]** and YC9 **[4]**. Reprinted and adapted with permission from Harmatys et al., Tuning pharmacokinetics to improve tumor accumulation of a prostate-specific membrane antigen-targeted phototheranostic agent, Copyright 2018, American Chemical Society 64.

In some cases, achieving complete resection of tumor tissue is challenging. For example when lymph nodes or positive tumor margins are located in close proximity to surrounding healthy tissue (e.g. nerves, bladder) 4. These difficult to resect tumor lesions can potentially be eradicated by targeted photodynamic therapy (tPDT) 57. The three components needed for tPDT are a light, oxygen and a photosensitizer. Upon activation, the photosensitizer undergoes an oxygen mediated photochemical process producing reactive oxygen species (ROS) which results in specific cellular damage of the target cells 61. In addition, tPDT may even lead to systemic immunity due to destruction of tumor cells inducing an anti-tumor immune response 57. As PSMA-targeted tracers with a photosensitizer are designed to accumulate in PCa lesions and the light (normal or laparoscopic 680nm laser) can be focused to the tumor site as well, tPDT is highly precise. Potentially, it enables therapy with minimal side effects 57. The first PSMA-targeted photosensitizer conjugates consisted of small molecule PSMA inhibitors coupled to porphyrin dyes. With these conjugates, including Ppa-CTT-54 **[1]** and LC-Pyro **[2]** (Figure 7A) feasibility of porphyrin-mediated PSMA-targeted PDT was shown *in vivo* (Figure 7B-C) 61-64. Further analysis showed caspase pathway activation, cleavage of polyADP-ribose polymerase (PARP), DNA fragmentation and rapid cytoskeletal disruption, leading to apoptosis and/or necrosis 61. Next to the porphyrin dyes, most studies have focused on small molecule PSMA inhibitors coupled to the photosensitizer IRDye700DX (690nm), including the tracers called PSMA-1-IR700 **[3]** and YC9 **[4]** (Figure 7D) 65, 66. *In vivo* light irradiation using these PSMA-IRDye700DX tracers led to significant tumor size reduction of PC3-PIP PSMA-positive s.c. tumors compared to the PSMA-negative tumors, without apparent off-target toxicity. Moreover, a delay in tumor growth and an increase in mean survival were observed, demonstrating the potential to effectively inhibit PC3-PIP tumor progression with these tracers. Next, Watanabe *et al*. conjugated IRDye700DX to the humanized J591 anti-PSMA antibody and its fragments 67. They showed significant tumor growth delay and prolonged survival in mice bearing PSMA-positive PC3 tumors, again indicating the feasibility of IRDye700DX mediated tPDT. The first study to describe tPDT with multimodal IRDye700DX tracers for PCa detection, resection and irradiation was performed by Lütje et al 68. They developed the theranostic PSMA targeting agent [^111^In]In-DTPA-D2B-IRDye700DX by conjugating DTPA and IRDye700DX (Figure 3B, **[11]**) to the murine anti-PSMA antibody D2B and subsequently labeled it with ^111^In. In 5 mice, intraoperative NIRF imaging could be used for guided resection of subcutaneous LS174T-PSMA tumor xenografts, demonstrating the applicability of the multimodal conjugate for intraoperative image-guided resection of tumor lesions. tPDT treatment of LS174T-PSMA tumor bearing mice using this tracer caused significant tumor growth delay, as tumors in the control group reached a size of 500 mm^3^ in 27.7 days (range: 19-42 days), while treated tumors reached this size after 50.4 days (range: 23-80 days). In addition, median survival of the treated mice (47 days) was significantly longer than that of the untreated mice (27days). This study provided the first proof-of-principle that multimodal [^111^In]In-DTPA-D2B-IRDye700DX can be used for pre- and intraoperative detection of PSMA positive tumors with radionuclide- and fluorescence-imaging, surgical guidance, and PSMA-targeted PDT. PSMA-targeted PDT for PCa therapy is still in its infancy, no clinical proof of concept studies have been performed yet. However, despite the fact that a lot of translational steps towards the clinic still need to be taken, PSMA-targeted PDT has a high potential to become a valuable therapeutic option especially to remove remaining tumor cells in PCa patients during and after surgery.

## Discussion

In order to image PCa cells via PSMA-expression a suitable tracer is required. Important requirements for this are: 1) High uptake in PSMA-positive tumor tissue, 2) good tumor retention up to the moment of surgery and 3) low uptake in background tissue. Sufficient tumor-to-background ratios are key for targeting and imaging of prostate cancer. Given the variety of available and clinically used PSMA tracers, it is difficult to reach consensus on which ligand, radionuclide and/or fluorophore combination is best suited for a particular application. Still, PSMA-targeting ligands should be designed to meet a specific clinical need. In case of intraoperative PCa detection several factors are important as will be discussed below.

### Type of targeting agent

A lot of effort has been put into finding the optimal PSMA-binding small molecule, antibody fragment or whole antibody. Important properties of these PSMA-binding tracers are specificity for PSMA and sufficient accumulation and retention in the tumor to allow intraoperative detection. The first described PSMA binding tracers are monoclonal antibodies (mAbs) 69. Well known high affinity anti-PSMA mAbs include the J415, J533 and J591 series, which bind to the extracellular domain of PSMA 69, 70. These mAbs hold promise for PCa detection and therapy. The main advantage of mAbs is the high absolute uptake in the tumor, caused by their high affinity and long circulatory half-life. In addition, compared to the renal clearance of small molecules, the hepatic clearance of mAbs leads to less background signal in the surgical field of PCa. However, because of their large size, accumulation in solid tumors can be slow (taking up to days) and high non-specific uptake in PSMA-negative tumors is observed, presumably as a result of the EPR effect 67. Despite the long circulating plasma half-lives, high uptake in normal tissue, and low tumor-to-normal tissue ratios, the high tumor uptake of these mAbs could be essential in NIRF imaging and tPDT, where a sufficiently high amount of tracer in the tumor is essential for imaging sensitivity and therapeutic outcomes 11, 71.

To overcome the disadvantages of mAbs and their derivates, small molecule PSMA-binding motifs with improved pharmacokinetics were developed 11, 72. The small size of the tracers could improve penetration of the dense tumor tissue, which is advantageous from both the diagnostic and the therapeutic point of view 57. The most commonly used small molecule PSMA tracers have glutamate-urea-lysine based PSMA-binding motifs 19, 72. They are structurally most similar to the normal PSMA substrate (NAAG) and therefore exhibit the best PSMA-binding properties 20. Despite the above mentioned advantages of small molecules, the use of small molecule PSMA binding motifs also poses several challenges. First, high background signal from the kidneys and bladder, due to the renal clearance of the small molecules, might hamper accurate intraoperative detection of PCa in the vicinity of these organs. Second, small molecules may have a lower absolute tumor uptake (in terms of %ID/g) when compared to mAbs, which could lead to concentrations that are too low to detect, visualize and treat with radiodetection, NIRF imaging and tPDT, respectively. Last, addition of imaging moieties and linkers can lead to major differences in affinity, pharmacokinetics and tumor uptake of the tracer. Nonetheless, multiple recent studies have shown that these challenges can be overcome and that, by creating NIRF/multimodality probes, the binding properties and pharmacokinetics of small molecule PSMA-ligands can even be improved 34, 54, 59. In general, poor vascularization and heterogeneity of the disease could impede PSMA-targeting of both mAbs and small molecules for surgical guidance and treatment, leading to false negative detection rates. Nonetheless, PSMA is highly expressed on more than >90% of all lesions 12. Next to PSMA-targeting agents, other tracers not discussed in the current review have the potential to improve the surgical treatment of PCa. These include for example folate or androgen receptor targeting agents, sodium fluoride (NaF) tracers and amino acid (mostly leucine) analogs 73, 74.

### Type of radionuclide

In addition to choosing the optimal tracer for PSMA targeted intraoperative detection of PCa, it is of utmost importance to choose the most suitable imaging moieties. The most commonly used radionuclides for intraoperative gamma probe based PCa detection are ^99m^Tc and ^111^In, which have several main advantages. First, besides intraoperative detection, these γ-emitters can be used for pre- and post-operative SPECT imaging. Second, ^99m^Tc and ^111^In have half-lives of 6 hours and 2.8 days respectively, leading to sufficient time for preoperative SPECT scanning, multiple-hour and/or next day surgeries. The 6-hour half-life of ^99m^Tc is better compatible with the fast pharmacokinetics of small molecule PSMA-tracers and could therefore be preferred over ^111^In. Last, the availability of the radionuclides is important. ^99m^Tc is readily available in many hospitals, leading to a high availability even in regional hospitals 21. In addition to its availability, ^99m^Tc often is preferred over ^111^In because of its lower production costs, and shorter half-life, leading to lower radiation exposure to both patients and nuclear medicine personnel. Moreover, ^99m^Tc emits lower energy gamma rays of 140 keV, that are preferred for collimation, and a greater activity can be administered per patient resulting in higher photon yields and better quality SPECT images 21, 75, 76.

Beside low-energy gamma emitting radionuclides (e.g. ^99m^Tc and ^111^In), RGS could also be performed using positron emitters such as ^68^Ga or ^18^F 77. However, for the detection of high-energy 511 keV gamma emissions a dedicated gamma-detection probe is needed with thicker shielding and/or a longer collimator 28, which results in drastically increased dimensions and overall weight of the probe. This probe will generally not fit through a standard trocar used during laparoscopic and robot-assisted surgery. As the current surgical treatment of patients with PCa is shifting towards a minimally-invasive setting, use of positron-emitting isotopes for RGS might therefore be limited 28, 38. Moreover, a recent study by Orsaria *et al*. reported poor performance of [^18^F]-FDG gamma probe-guided surgery in evaluating axillary lymph nodes in breast cancer patients, because of high background gamma levels from local and distant parts of the body 78.

### Type of fluorophore

In general, dyes that have emission wavelengths in the near-infrared (NIR) region of the spectrum (650-900 nm) are employed for intraoperative imaging. Main advantages of using these NIR dyes are a superior tissue penetration depth, reduced interference from the presence of blood in the surgical field and low auto-fluorescence of the tissue in the NIR region 47. Indocyanine green (ICG, emission peak at 830 nm) is one of the few fluorophores approved for intraoperative clinical use by the Food and Drug Administration (FDA). However, ICG by itself is non-target specific and in oncology mostly used to map tumor-draining lymph nodes 79. A number of other NIR dyes have shown great promise in preclinical *in vivo* imaging studies, including Cy5.5, Cy7, Dylight800, ICG derivatives and IRDye800CW. In a study to determine which of these dyes shows the best *in vivo* properties, all dyes were labeled to the same PSMA-binding small molecule. Both the Cy7- and IRDye800CW-ligands showed superior PSMA-specific tumor uptake, internalization and tumor-to-background ratios. Moreover, the IRDye800CW-ligands displayed a much higher fluorescent intensity 47. In another head-to-head comparison of different fluorescent dyes, IRDye800CW and IRDye700DX were found highly suitable for fluorescence imaging due to their brightness and photostability 80. The IRDye800CW is often preferred over IRDye700DX for imaging purposes, as it is higher in the NIR spectrum, has a higher intensity and is a smaller and more stable molecule under *in vivo* conditions. Nonetheless, IRDye700DX has the major advantage of being a potent photosensitizer. This means that, during surgery, theranostic IRDye700DX tracers can also be used to eradicate the remaining unresectable cancer cells using tPDT 81. Hence, different dyes with varying characteristics are suitable for intraoperative imaging and the exact choice of dye will also highly depend upon the on the specifications of the camera used for detection in the operating room.

### PSMA-guidance for PCa detection in minimally invasive surgery

With the rapid growth of minimally-invasive laparoscopic and/or robot-assisted PCa surgery, there is a need for image-guidance technologies suitable for these procedures. One important development in fluorescence-guided robotic surgery is the introduction of the Intuitive Surgical's Firefly™ imaging technology (Novadaq Technologies, Mississauga, ON, Canada) integrated in the da Vinci® robot.^53^, ^82^ This surgical system is equipped with filters that are optimized for detection of indocyanine green (ICG, peak emission wavelength 830 nm), but could also be used for the detection of IRDye800CW (peak emission wavelength 792 nm). For detection of this IRDye800CW, proof of concept has been demonstrated with the PSMA-ligand YC-27 53. However, no filters for the detection of fluorescent dyes in other spectra are available yet. This hampers the use of photosensitizer-conjugated theranostic PSMA-ligands (peak emission wavelength ~650-700 nm) in minimally invasive fluorescence-guided surgery 64, 65.

In RGS, laparoscopic gamma probes are used for *in vivo* and *ex vivo* measurement of the radiosignal in minimally-invasive surgical procedures. However, these probes have limited maneuverability *in vivo* and therefore hamper the detection of low activity lesions that are located near high background areas such as the kidneys 83. To overcome this problem, van Oosterom *et al*. have developed a DROP-IN gamma probe with an effective scanning direction range between 0-180°, that was proven to be a valuable tool for robot-assisted RGS procedures 83, 84.

### Opportunity for PSMA-guided surgery in other malignancies

PSMA is expressed on the neovascular endothelium of numerous non-prostate solid tumors 85-88. Chang *et al.* studied PSMA-expression in the neovasculature of 15 non-prostate tumors using five different PSMA mAbs. In this study a strong neovascular PSMA immunoreactivity was described in many tumors, including clear cell renal cell carcinoma, colonic adenocarcinoma, glioblastoma multiforme, non-small cell lung carcinoma and breast carcinoma 86. Targeting PSMA-expression in the tumor associated neovascular endothelium could therefore enable intra-operative imaging of a variety of malignancies. Despite the fact that intra-operative PSMA-imaging studies of these other malignancies were not described in literature yet, successful PSMA-based pre- and post-operative imaging support the idea that targeted intra-operative PSMA-imaging, to prevent positive surgical margins, could be extended beyond PCa. For instance, in a pilot study by Sasikumar* et al.* the feasibility of pre- and postoperative imaging of gliomas using^ 68^Ga-PSMA-11 PET/CT was investigated 89. From the 10 patients scanned preoperatively, 9 were positive and proven to be a true recurrence in post-surgical pathological analysis. In 3 patients scanned immediately after surgery, the presence or absence of disease could be identified on the ^68^Ga-PSMA-11 scan and correlated with conventional MRI imaging. Furthermore, in a recent study by Meyer *et al.* the added value of pre-operative PSMA-based ^18^F-DCFPyL PET/CT imaging was suggested in patients with oligometastatic renal cell carcinoma 90. In 28.6% of the patients a total of 12 more lesions were identified on ^18^F-DCFPyL PET/CT compared to conventional imaging, leading to detection rates of 88.9% and 66.7%, respectively.

However, in other cancer types absolute tumor uptake of the PSMA-tracers is lower compared to PCa. Expression of PSMA is only present on the neovasculature, with little to no expression on the tumor cells and the normal vascular endothelium, possibly leading to lower detection rates. Therefore, PSMA-based imaging is most suitable for tumors with a high rate of neovascularization.

## Conclusion and Future Perspective

The detection and removal of positive resection margins, small tumor lesions and micro-metastasis still poses major challenges in the surgical management of patients with PCa. Unfortunately, at the moment surgeons have to choose between complete oncological resection, which often leads to debilitating side effects, and nerve-saving operations focused on functional outcomes, with a higher chance of positive resection margins as a consequence 9. With the development of highly specific PSMA ligands, intraoperative PCa detection and the essential margin detection becomes available. Given the variety of the developed PSMA tracers, together with the fact that no consensus has been found on which tracer is most suitable for intraoperative use, in this manuscript we discussed considerations for the design of such PSMA tracers.

It is debatable which type of tracer is best suited for intra operative use, as both mAbs and small molecule PSMA tracers each have their own advantages, including high tumor uptake of mAbs and high specificity and fast pharmacokinetics of small molecule PSMA tracers. Modifications in small molecule PSMA-tracers, including linker length and addition of multiple negative charges, were found to alter the binding affinity of the PSMA ligands causing improved targeting characteristics and reduced background signals. At present, ^99m^Tc and ^111^In-labeled small molecule PSMA ligands, already used in a clinical studies, seem to be the most promising tracers for radioguided surgery. Currently, for PCa surgical removal of cancerous tissue is performed without any fluorescence image guidance. In the up-and-coming fluorescence-guided surgery field, NIR dye labeled PSMA ligands show encouraging results. In addition, photosensitizers like IRDye700DX could be used for tPDT of residual tumor lesions. Hence, use of such a theranostic dual-labeled PSMA ligand allows for imaging, resection and local killing of PCa cells.

Presumably the most promising developments in the intraoperative field are the dual-labeling strategies that allow for both acoustic and visual detection of PSMA-expressing tumor lesions, micro-metastases and positive resection margins. Together, these multimodal strategies empower guided total resection of PCa tumors. Unfortunately, so far, fluorescent and dual-labeled PSMA-targeting tracers have only been investigated in a preclinical setting. In order to move the field forward, it is essential that proper prospective clinical studies are carried out measuring the additional value of multimodal image guided surgery compared to conventional surgery techniques. Clinical trials comparing these two treatments should be performed and main outcomes of these studies should include comparison of toxicology, complications, progression free survival, overall survival and quality of life of the PCa patients.

## Figures and Tables

**Table 1:** Overview of PSMA ligands for intraoperative PCa detection

Reference	Year	PSMA ligand	Radio-nuclide		Fluoro-phore*	Research status		Main results
Radioguided surgery								
Maurer et al. 35	2015	lysine-urea-glutamate		^111^In (γ)	-		Clinical feasibility, 5 patients	- Identification of additional positive lesions not detected during preoperative PET/CT imaging
Schottelius et al. 22	2015	lysine-urea-glutamate		^111^In (γ)	-		Exemplary patient	- Radioguided resection of PSMA-positive lesions
Robu et al. 21	2017	lysine-urea-glutamate		^99m^Tc (γ)	-		Two exemplary patients	- Successful detection and resection of radiosignal-positive lesions
Maurer et al. 23	2018	lysine-urea-glutamate		^99m^Tc (γ)	-		Retrospective analysis, 31 patients	- Comparison of radioactive rating with histopathological analysis; specificity 93%, sensitivity 83.6%
Rauscher et al. 36	2017	lysine-urea-glutamate		^111^In (γ) + ^99m^Tc (γ)	-		Clinical follow up, 55 patients	- PSA level reduction > 50% in 44 (80%), > 90% in 29 (53%) patients
Horn et al. 24	2017	lysine-urea-glutamate		^111^In (γ) + ^99m^Tc (γ)	-		Clinical follow up, 59 patients	- PSA < 0.2 ng/mL in 67% of patients
Fluorescence-guided surgery								
Humblet et al. 43	2005	GPI		-	IRDye78 (771-796 nm)		Preclinical, s.c. LNCaP	- NIR fluorophore conjugation improved PSMA-affinity over 20-fold
Wang et al. 44	2014	PSMA-1		-	IRDye800CW (778-794 nm) + Cy5.5 (675-694 nm)		Preclinical, orthotopic PC3-PIP	- Pharmacokinetics highly dependent on the conjugated fluorophore
Bao et al. 34	2017	lysine-urea-glutamate		-	Cy5.5 (675-694 nm), Cy7 (753-775 nm) + ZW800+3C (774-789 nm)		Preclinical, s.c. LNCaP	- Physicochemical properties of fluorophores drastically alter characteristics of ligands
Kularatne et al. 51	2018	DUPA		-	S0456 (788-800 nm)		Preclinical, s.c./orthotopic 22Rv1/LNCaP	- Sub-nanomolar concentration sufficient to visualize small lesions. - Retention of fluorescent signal in PSMA^+^ tumors > 48 hours
Kelderhouse et al. 52	2013	DUPA		-	AF647 (650-665 nm), Dylight680 (692-712 nm) + IRDye-800CW (778-794 nm)		Preclinical, intracardial injections 22Rv1	- Complete resection of metastasis with minimal contamination from healthy tissue
Kovar et al. 1	2014	YC-27		-	IRDye-800CW (778-794 nm)		Preclinical, s.c. 22Rv1	- High tissue contrast and sufficient tumor delineation at doses as low as 0.25 nmol
Neuman et al. 53	2015	YC-27		-	IRDye-800CW (778-794 nm)		Preclinical, s.c. PC3-PIP	- 0% recurrences when resected with NIRF-guidance compared to 40% in control white-light mice
Multimodal-guided surgery								
Banerjee et al.^58^	2011	lysine-urea-glutamate		^111^In (γ)	IRDye-800CW (778-794 nm)		Preclinical, s.c. PC3-PIP	- Multimodal visualization, delineation and high uptake of the tracer, 16.4±3.7 %ID/g**
Baranski et al. 54	2017	PSMA-11		^68^Ga (β^+^)	IRDye-800CW (778-794 nm)+ Dylight800 (777-794 nm)		Preclinical, s.c. LNCaP + healthy pigs	- Conjugation of IRDye800CW (13.6 ± 3.7 %ID/g) and DyLight800 (15.6 ± 5.5 %ID/g) increased specific tumor uptake** - Ligands enabled radical prostatectomy under fluorescence-guidance in pigs
Schottelius et al. 59	2018	lysine-urea-glutamate		^68^Ga (β^+^)	Sulfo‐Cy5 (646-662 nm)		Precinical, s.c. LNCaP	- PSMA-specific uptake in tumor (4.5 ± 1.8 %ID/g)** - Tumor/background ratios at 1 h p.i. of 2.1 and 9.6 for blood and muscle.
								

* Fluorescent wavelengths are indicated as excitation maximum-emission maximum in nm.** Note that PSMA expression levels differ between tumor models used. Therefore, uptake values (%ID/g) cannot be directly compared. **Abbreviations**: p.i.; post injection, s.c.; subcutaneous, PSMA; Prostate specific membrane antigen, h; hours, %ID/g; Percentage injected dose per gram, NIRF; Near-infrared fluorescence.
